# Behavioural science to improve effectiveness of HIV programmes, South Africa

**DOI:** 10.2471/BLT.21.285626

**Published:** 2021-09-28

**Authors:** Gavin George, Brendan Maughan-Brown, Harsha Thirumurthy

**Affiliations:** a Health Economics and HIV and AIDS Research Division (HEARD), University of KwaZulu-Natal, Westville Campus, Durban 4041, South Africa.; b Southern Africa Labour and Development Research Unit (SALDRU), University of Cape Town, Cape Town, South Africa.; c Perelman School of Medicine, University of Pennsylvania, Philadelphia, United States of America.

Human behaviour plays a central role in determining the effectiveness of biomedical interventions. Understanding how people make decisions and devising appropriate policy responses can therefore be instrumental in bridging the efficacy of interventions in randomized trials and their effectiveness during their implementation in routine care settings. South Africa’s response to the human immunodeficiency virus (HIV) epidemic provides insights into how using behavioural science can increase the effectiveness of biomedical interventions. 

South Africa has the world’s largest HIV epidemic, with 7.8 million people living with HIV and an adult HIV prevalence of 19% (7 500 000/~39 200 000).^1^ Over the past two decades, however, South Africa has made substantial progress in combating HIV. Testing, voluntary medical male circumcision and antiretroviral therapy (ART) have been rapidly scaled up. With expanded service delivery and accompanying behavioural interventions, over 92% (7 200 000/7 800 000) of people living with HIV are aware of their HIV status.^1^

However, many obstacles remain to ending South Africa’s HIV epidemic. Only 72% (5 600 000/7 800 000) of people living with HIV have initiated ART – a missed opportunity for protecting their health and preventing onward transmission. The coverage of HIV services is also much lower among men than women, while HIV incidence remains extremely high among adolescent girls and young women.^1^

Human behaviour is a key factor in determining the effectiveness of HIV programmes. These programmes have had to overcome various behavioural challenges, including low HIV testing rates among key populations, poor linkage to and retention in HIV care and slow uptake of voluntary medical male circumcision. Here we discuss the behavioural science principles that explain why such challenges arise and highlight policies and interventions that have sought to address these challenges.

## Understanding behavioural science 

People rely on heuristics and exhibit biases when making decisions. Heuristics, which are sometimes referred to as rules of thumb or mental shortcuts, are experience-based strategies that can simplify decision-making but lead to cognitive biases. In addition, present bias can explain why people delay seeking HIV services despite knowing their benefits and having the intention to use the services. The benefits of using these services are achieved in the future (improved health), whereas the costs (out-of-pocket expenses, time costs, hassle factors such as waiting in clinics and social stigma) are immediate, obvious and highly influential in people’s decision-making. People also display substantial inertia when it comes to investing in their own health, often opting to do nothing rather than taking a proactive decision, such as going for an HIV test – that is, status quo bias. People’s behaviour also tends to conform to prevalent social norms; for example, the motivation to get tested may be low if peers are not testing.

Difficulty in forming accurate risk perceptions or processing health information can further affect behaviour in ways that may be harmful to health, whether through riskier sexual behaviour or underutilization of HIV services. Affective factors such as mood, emotions and stress further influence people’s decisions.

Finally, scarcity of resources, particularly money, food or family support, can affect behaviour and cognitive ability, resulting in poorer decisions about personal health and welfare. Such shortages can also amplify biases in decision-making, increase reliance on mental shortcuts and hamper the ability to process health information. At the extreme, people focus all their attention on their immediate needs and de-prioritize health behaviours, which could be detrimental to future health.

## Response to behavioural challenges

South Africa’s HIV response has benefited substantially from innovations in service delivery that directly address some of the behavioural barriers mentioned above. Given the importance of status quo bias, changes in the default way that HIV services were offered to individuals increased the likelihood of people using those services, without limiting individual autonomy. For example, provider-initiated testing at health facilities resulted in an increase in testing rates.^2^ Since present bias is influential in decision-making, bringing HIV testing services closer to people through community-based testing campaigns, home-based testing or workplace testing has contributed to higher testing coverage. Such service delivery models counter the effect of present bias by reducing the immediate economic costs associated with getting an HIV test. These models also reduce the psychological costs associated with testing, as stigma can be reduced if many people in a community or workplace are participating together. For example, men are less likely to use facility-based health services; therefore, having alternative service delivery models, along with innovations such as HIV self-testing, have proven to increase service uptake.^3^ Finally, media campaigns and testimonies from prominent people living with HIV have sought to normalize living with HIV, and address the stigma and fear of discrimination associated with HIV.^4^

In 2016 and 2017, South Africa implemented the World Health Organization’s guidelines on universal test and treat and same-day initiation of ART, while continuing to expand access to services in community-based settings. These policy changes and the implementation of task-shifting strategies – which included the expansion of nurses’ roles to include ART initiation and re-prescription – have contributed to expanding ART coverage among people living with HIV.^5^ The introduction of differentiated ART models led to a shift from a one-size-fits-all to a patient-centred approach, enabling stable patients on ART to access their medication with fewer and faster visits to health facilities or community-based services.^6^ Differentiated service delivery has proven to be effective in ensuring the contextual needs of patients are met, and has mitigated the potential negative impact of the coronavirus disease 2019 (COVID-19) on HIV retention in care rates.^7^ The ability to obtain prescriptions covering several months or community ART delivery has assisted patients when normal health-care services have been crowded out by COVID-19 care services, or movement restrictions made access to health facilities challenging.^7^

South Africa has also invested in strategies aimed at increasing demand and access to biomedical HIV prevention interventions. Demand for voluntary medical male circumcision was promoted through several behavioural interventions, including messages emphasizing that women preferred circumcised partners. The roll-out of comprehensive sexuality education in schools has had positive effects on ideational factors, namely changes in knowledge and stigma.^8^ Bringing health services to schools has further led to increased uptake of HIV testing and access to condoms among adolescents.^9^

Several studies in South Africa and elsewhere have also shown that providing incentives can increase uptake of various HIV services, including HIV testing and medical male circumcision. Examples of incentives have included monetary and lottery-based rewards, food vouchers and household items. While few HIV programmes have scaled up incentives, many have provided food or transport vouchers to reduce the costs associated with access.^10^ Several unconditional and conditional cash transfer programmes have further sought to address structural factors that affect HIV outcomes, including school attendance, though these have had mixed results.^11^

## Looking ahead

Using behavioural insights in the HIV response remains essential as South Africa and other high HIV-burden countries seek to expand coverage of HIV services among key and priority populations, increase retention in HIV care and expand use of prevention options.

Behavioural science research and practice have shown potential to influence human behaviour through behavioural nudges, that is, low-cost interventions that change the presentation of choices and alter people’s behaviour in predictable ways, without restricting freedom of choice.^12^ Examples include changes in the way services are delivered, increased use of mobile health technology or provision of small incentives. Considerable room for interventions on the supply side remains, as behavioural science approaches can be used to promote job satisfaction and performance of health-care providers. Additionally, behavioural science principles can explain why policy-makers and programme implementers initially underestimate the importance of human behaviour when scaling up HIV services. Policy-makers can exhibit optimism bias and the associated appraisal bias, whereby they focus on the best-case scenario and overestimate the extent to which people are driven by the health benefits of HIV services. Consensus bias can also lead policy-makers to believe their own judgements and behavioural choices would be replicated by those for whom the services are targeted. Recognition of factors that affect decision-making is therefore critical in adjusting policy and programmes, ensuring they address these impediments to maximize their effectiveness.

The introduction of new biomedical prevention or treatment technologies is often accompanied by high levels of optimism and expectation around projected demand and effectiveness. However, without adequate investment in the application of behavioural science principles in the design and implementation of these programmes, uptake and subsequent effectiveness is likely to be overestimated. A similar focus on demand creation is necessary, as promising new prevention products such as injectable pre-exposure prophylaxis and a potential vaccine become available.

South Africa’s HIV response offers examples of how policies and strategies have addressed behavioural challenges. However, ensuring that programmes are designed using behavioural science principles during the inception stage is key to increasing efficiency and to creating enabling environments in which people can make healthier decisions. While most behavioural nudges and low-cost interventions to address these challenges will not eliminate the structural barriers that influence human behaviour, they can significantly offset the consequences of contextual challenges and promote positive behaviour.
